# Clinical implications and immune implications features of TARS1 in breast cancer

**DOI:** 10.3389/fonc.2023.1207867

**Published:** 2023-08-11

**Authors:** Zhengwei Gui, Piao Liu, Dong Zhang, Wanju Wang

**Affiliations:** ^1^ Tongji Hospital, Tongji Medical College, Huazhong University of Science and Technology, Wuhan, China; ^2^ Department of Breast and Thyroid Surgery, Tongji Hospital, Wuhan, Hubei, China; ^3^ Department of General Surgery, Hubei Provincial Hospital of Integrated Traditional Chinese and Western Medicine, Wuhan, Hubei, China

**Keywords:** TARS1, breast cancer, cell proliferation, immune infiltration, clinical feature

## Abstract

**Background:**

There has been an increase in the number of women suffering from breast cancer in recent years, and discovering new therapeutic targets and efficacy predictive markers is critical for comprehensive breast cancer treatment.

**Methods:**

First, we used bioinformatics methods to analyze TARS1(encoding cytoplasmicthreonyl-tRNA synthetase) expression, prognosis, and clinicopathological characteristics in TCGA database breast cancers, and then we collected breast cancer specimens from our center for validation. TARS1 was then subjected to GSEA (Gene Set Enrichment Analysis) enrichment analysis, GO/KEGG pathway enrichment analysis, and breast cancer immune infiltration characterization. As a last step, we examined TARS1’s effects on breast cancer cell behavior with cellular assays.

**Results:**

The overexpression of TARS1 has been found in several malignant tumors, including breast cancer, and has been linked to poor prognoses. Breast cancers with large primary tumors and negative hormone receptors are more likely to overexpress TARS1. Overexpression of TARS1 promotes the infiltration of T cells, such as Tregs and Th2s, while inhibiting the infiltration of NK cells and CD8+ T cells, which are anticancer cells in breast cancer. TARS1 was also found to be co-expressed with the majority of immune checkpoint-related genes, and breast cancer with TARS1 overexpression responded better to immunotherapy. By knocking down TARS1, breast cancer cells were prevented from proliferating and invading, as well as exhibiting other malignant biological properties.

**Conclusion:**

According to our study, TARS1 may be an oncogene in breast cancer and may be a biomarker of efficacy or a target of immunotherapy in breast cancer.

## Introduction

Globally, breast cancer (BC) is the most common malignancy among women, threatening the health of more and more individuals every day (1). The promotion of comprehensive breast cancer treatment, which includes surgical treatment, chemotherapy, radiotherapy, endocrine therapy, and targeted therapy, has considerably improved the prognosis of BC patients (2). In particular, there is still no effective treatment available for triple negative breast cancer, which is a pathological form of breast cancer (3). Therefore, discovering new breast cancer oncogenes and developing new therapeutic targets are critical for improving the prognosis of breast cancer. The efficacy of various therapy regimens for different forms of breast cancer varies substantially (4). As a result, distinguishing between the major breast cancer pathological stages and other clinicopathological aspects impacted by the causal genes is critical for accurate breast cancer treatment (5, 6).

Immunotherapy is regarded as the new treatment with the greatest potential to cure cancer at its source (7); nonetheless, its efficacy in breast cancer needs to be improved. Discovering novel immunotherapy efficacy prediction biomarkers will allow for focused immunotherapy for breast cancer. Precision therapy could lower national health care investment, which is especially essential for many developing countries, according to health economists (8).

The threonine-tRNA synthetase (TARS) is an aminoacyl-tRNA synthetase that plays a key role in protein synthesis. Aminoacyl-tRNA synthetases (aaRS) are housekeeping proteins that catalyze the attachment of tRNAs to homologous amino acids, hence providing aminoacyl-tRNA building blocks for ribosomal protein synthesis (9). Mammalian cytoplasmic and mitochondrial protein synthesis each have their own set of aaRSs, whereas TARS1 and TARS2 encode eukaryotic cytoplasmic and mitochondrial threonine-tRNA synthetases (ThrRSs) (10, 11). TARS1 has been demonstrated to be important in muscle development and is released during inflammation to enhance endothelial cell migration and angiogenesis (12). TARS1 is also involved in the regulation of translation initiation, which helps to positively regulate vertebrate mRNA translation (13).There is evidence that TARS is upregulated in gastric cancer and is associated with poor outcome and metastasis (14), in endometrial cancer, TARS1 was associated with poor outcomes (15). As of yet, no clear understanding of TARS1’s role in breast cancer has been established.

Initially, we examined the expression of TARS1 in various cancers, including breast cancer, the impact of TARS1 on breast cancer prognosis and its association with clinicopathological characteristics of breast cancer patients using data from the TCGA database and GTEx database, and collected breast cancer specimens from our center for quantitative analysis and validation. Then, using GSEA analysis, GO/KEGG pathway enrichment analysis, and breast cancer immune infiltration analysis, the potential benefit of TARS1 for breast cancer treatment was investigated. As a final step, we assessed TARS1’s potential to predict immunotherapy effectiveness using TIDE (Tumor Immune Dysfunction and Exclusion), by analyzing its co-expression with immune checkpoint-related genes.

Breast cancer treatment is entering the precision therapy era, with several studies leading to new personalized medicines and biomarkers. Traditional indicators such as ER, PR, and Her-2 have improved the prognosis of breast cancer patients dramatically (16–19). When chemotherapy is used, commercial gene expression combinations like OncotypeDX and Mammaprint are the best prognostic predictors for ER-positive, HER2-negative, lymph node-negative breast cancer (20, 21). New prognostic indicators are also indicated in specific metastatic breast cancer scenarios. The NCCN advises testing for BRCA1/2 germline mutation status in each metastatic patient to anticipate the potential benefit of PARP inhibitor therapy (22, 23). For targeted therapy, they may additionally include MSI/MMR, TMB, and NTRK (24, 25). The discovery of novel biomarkers is critical for improving prognosis and lowering healthcare costs for breast cancer patients (26, 27).

## Materials and methods

### The collection and processing of data

We used R and Graphpad Prism version 8.0 to conduct all statistical analyses and visualizations. Data from the GTEx and TCGA databases were used to analyze breast cancer patients’ mRNA expression profiles. We have removed duplicate samples and those lacking clinical information. In total, there were 179 paracancerous tissues and 1065 breast cancer tissues. The survival curve data were obtained from the KM plotter website (28).

### Correlation and enrichment analyses

The TCGA-BRCA database was analyzed for gene co-expression, the FoldChange was arranged in descending order, the genes with P>0.05 were removed, and the top 300 genes were selected for GSEA enrichment analysis. The 324 genes with absolute FoldChange values greater than 1.5 and P<0.05 were selected for GO/KEGG analysis.

### Pathological sample collection and processing

We collected 76 breast cancer specimens from Tongji Hospital between September 2021 and February 2023. There were 24 pairs of fresh frozen tissues matched with paracancer, 21 pairs of paired paraffin-embedded tissues, and 32 cancer tissue specimens. A protocol for this study has been approved by the Ethics Committee of Tongji Hospital in accordance with the Helsinki Declaration (approval number TJIRB20221218). Fixation of tissues in 10% formalin, paraffin embedding, serial sectioning into 5 mm layers, dewaxing, rehydration, and microwave antigen repair were all carried out on the tissues. At 1 degree Celsius, the slides were incubated overnight with 1:200 dilutions of TARS1 antibody (AFFINITY, df2315). The secondary antibodies were incubated for 30 minutes at room temperature before being stained with the DAB substrate and then re-stained with hematoxylin. The quantitative immunohistochemical analysis was carried out using ImageJ and AI tools.

### extraction and quantitative real-time PCR: RNA

According to the manufacturer’s instructions, total RNA was extracted using the TRIzol reagent (Invitrogen, USA). DynaScience Biotechnology in China provided qRT-PCR primers, including those for TARS1 and GAPDH. The primer sequences: TARS1: forward - TGTGCCATTGAATAAGGA, reverse - CACCTTCATTATCAAGATAC (5’-3’). GAPDH forward - GGAGCGAGATCCCTCCAAAAT, reverse -GGCTGTTGTCATACTTCTCATGG. The PCR conditions were as follows: 95°C for 5 minutes; (95°C for 5 seconds, 60°C for 30 seconds) 40 amplification cycles. Relative expression levels were standardized to the internal control and computed according to the 2-ΔΔCT technique.

### Cell culture and treatment

Shanghai Institute of Cell Biology provided the MCF7, MDA-MB-231, MDA-MB-468, and SKBR3 human breast cancer cell lines. MDA-MB-468 cells were cultivated in RPMI-1640 media (Gibco, USA), whereas MCF7, MDA-MB-231, and SKBR3 cells were grown in DMEM medium (Gibco, USA). All media are supplemented with 10% fetal bovine serum. All cell lines were cultured at 37°C in a ThermoFisher incubator with 5% CO2. By employing STR to identify and compare all bought cell lines to reputable databases.

### CCK8 assay

Cells from each experimental group that were in the logarithmic growth phase and under good growth conditions were digested and resuspended in full culture medium. Proliferation of cells was determined according to the manufacturer’s instructions using the Cell Counting Kit-8 (Invitrogen, USA). A marker enzyme was used to measure the optical density at 450 nm.

### Colony-formation assay

During the 14-day culture period, 1000 breast cancer cells were injected into six-well plates. The medium was replaced every three days and the medium utilized for each of the cells was as previously described. We stained the cell colonies with crystal violet after they had been fixated in 4% polyacetal for 10 minutes, photographed, and counted.

### Transwell assay

Transwell chambers in 24-well plates are filled with 20,000 breast cancer cells each. Various cells were resuspended in serum-free medium, uniformly added to the upper chamber, and the lower well was filled with medium containing 10% fetal bovine serum. We wiped the top surface of the chamber after incubating the cells at 37°C for 24 hours. A 10-minute fixation process with 4% paraformaldehyde was followed by a 10-minute staining process with crystal violet on the bottom surface of the chambers. Counting and photographing migratory cells was done.

### Scratch test

Breast cancer cells in the log phase of growth were inserted in 24-well plates with IBIDI two-well culture inserts and incubated for 24 hours. Forceps were utilized to carefully remove the culture implants from the immaculate table. Each well received 1 mL of low-serum medium, upon removal of the inserts, the migration rate of cells was determined under a light microscope at 0 and 24 hours.

### Immune cell infiltration

Using the GSVA package [version 1.34.0] of R, immune cell infiltration in BC was examined (version 3.6.3). On ssGSEA, the outcomes were based. The classification of immune cells and references to earlier studies’ markers were made. According to the median TARS1 expression in TCGA BC samples, two groups were identified (high and low), This dataset includes RNAseq data (level 3) as well as clinical information on 1101 breast tumors. TIDE was used to predict likely immunotherapeutic responses. Removal of duplicate samples and removal of samples that do not contain clinical information.

## Results

### TARS1 expression analysis

**Figure 1:**
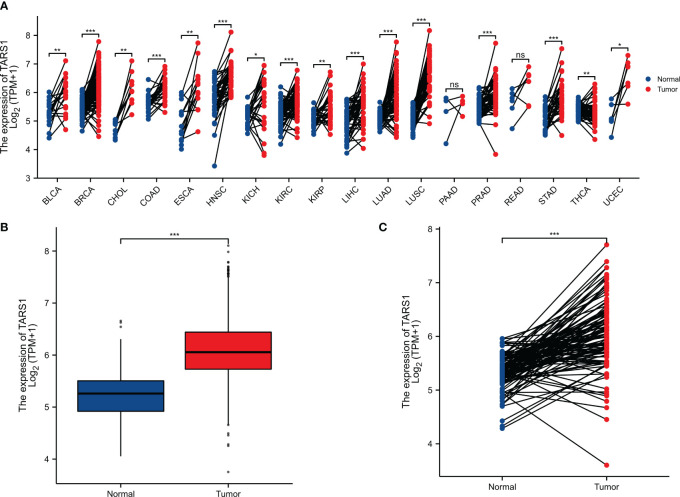
The expression difference of TARS1 in cancer tissue and normal tissue. **(A)** Expression of TARS1 in pan-cancer and adjacent normal tissues in TCGA and GTEx databases. **(B)** Expression of TARS1 in unpaired breast cancer samples in TCGA-BRCA database. **(C)** Expression of TARS1 in paired breast cancer samples in TCGA-BRCA database. Data were shown as mean ± SD. *p < 0.05, **p < 0.01, ***p < 0.001.

TARS1 was overexpressed in 15 cancers according to a pan-cancer study (**Figure 1A**). Contrarily, TARS1 was significantly overexpressed in breast cancer samples from both paired and unpaired individuals (**Figures 1B, C**).

### TARS1 expression and prognosis in breast cancer patients

**Figure 2:**
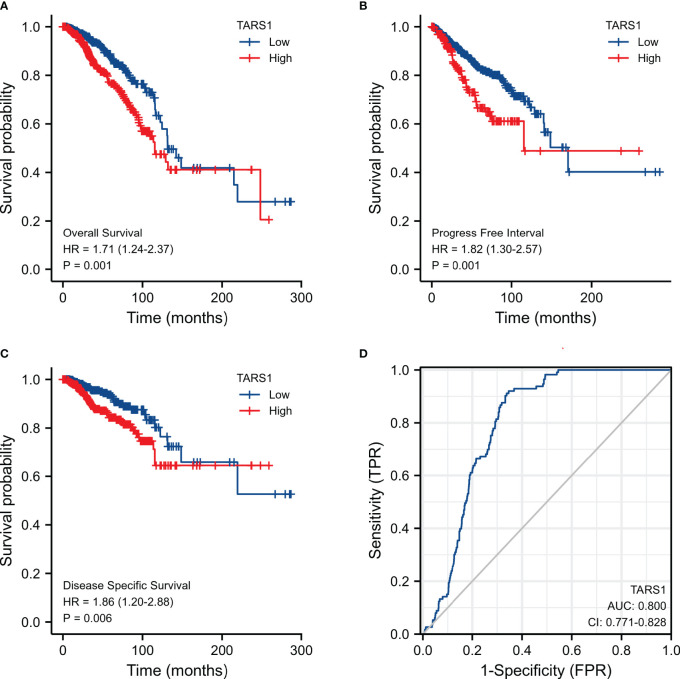
Expression of TARS1 and prognosis of breast cancer patients. **(A)** OS of breast cancer patients based on TARS1 expression level. **(B)** RFS of breast cancer patients based on TARS1 expression level. **(C)** DMFS of breast cancer patients based on TARS1 expression level. **(D)** ROC curve of TARS1.

KM plotter data showed that breast cancer groups with high TARS1 had significantly lower overall survival rates (HR=1.71, P=0.001), progress-free interval (HR=1.82, P=0.001), and disease-specific survival (HR=1.86, P=0.006). DSS (disease specific survival) decreased considerably (HR=1.86, P=0.006) (**Figures 2A–C**). TARS1-based ROC had an AUC of 0.800, CI: 0.771-0.828. (**Figure 2D**)

### Clinicopathological variables and TARS1 expression

**Figure 3:**
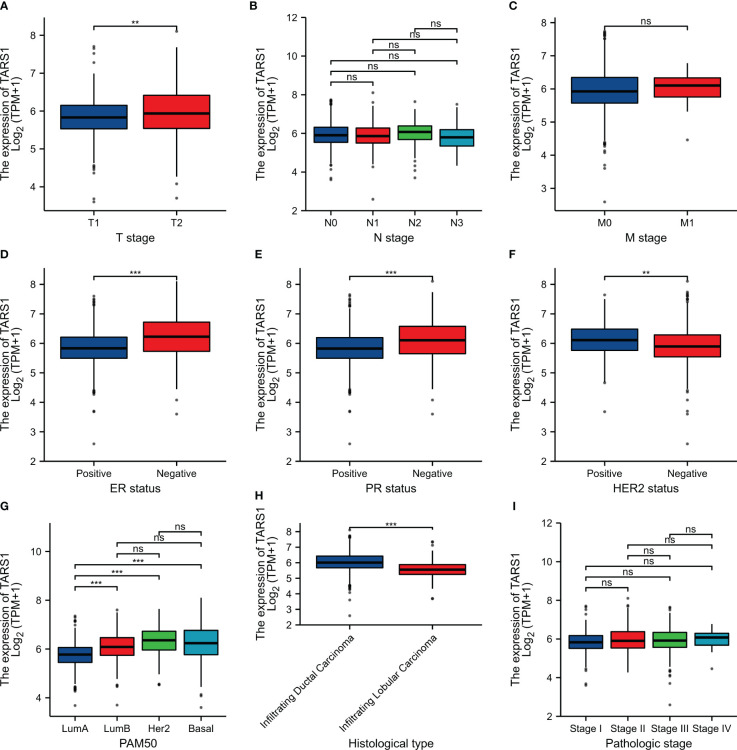
Relationship between TARS1 expression and clinicopathologic features of breast cancer patients in TCGA. Data are shown for **(A)** T stage; **(B)** N stage; **(C)** M stage; **(D)** ER status; **(E)** PR status; **(F)** HER-2 stage; **(G)** PAM50; **(H)** Histological type; **(I)** Pathologic type; *p < 0.05, **p < 0.01, ***p < 0.001. LumA, Luminal A; LumB, Luminal B; ER, estrogen receptor; PR, progesterone receptor; HER2, human epidermal growth factor receptor 2.

**Figure 4:**
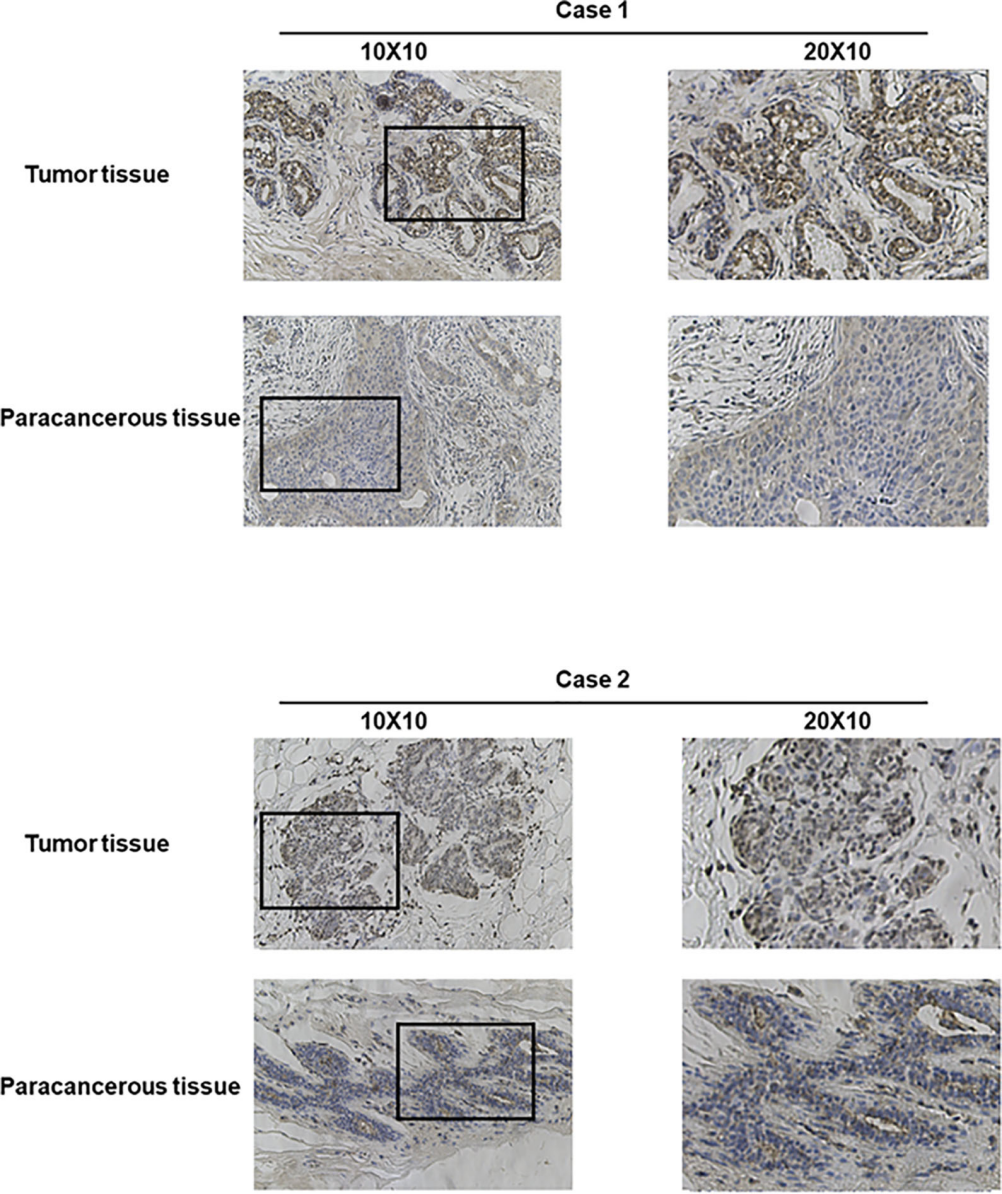
Representative images of TARS1 expression in breast cancer tissues and their matched paracancerous tissues. Original magnifications 40× and 100× (inset panels).

**Figure 5:**
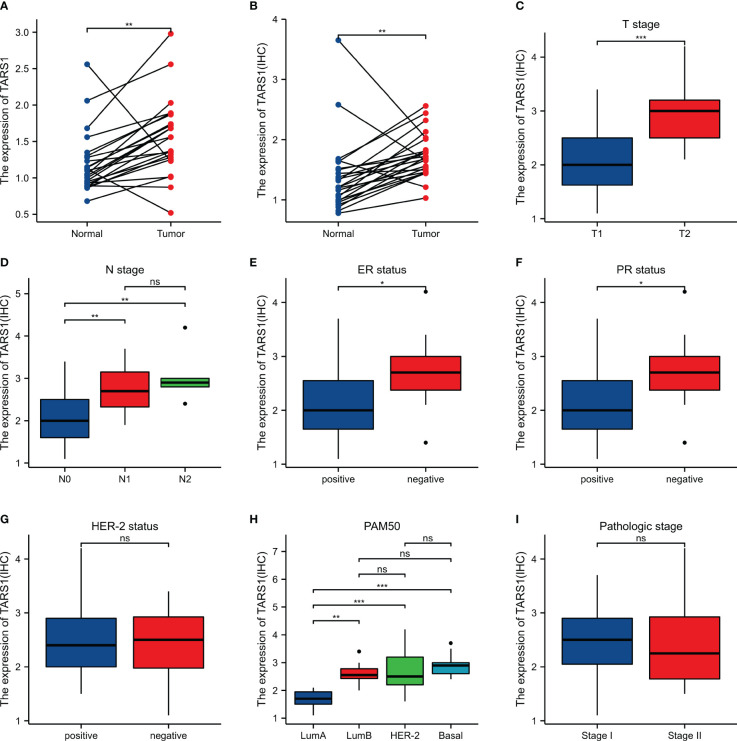
Expression and the relationship between TARS1 and breast cancer clinicopathologic features in our center. **(A)** mRNA levels of TARS1 in 24 pairs of fresh frozen specimens **(B)** Protein levels of TARS1 in 21 pairs of paraffin sections **(C)** T stage; **(D)** N stage; **(E)** ER status; **(F)** PR status; **(G)** HER-2 status; **(H)** PM50; **(I)** Pathologic stage; *p < 0.05, **p < 0.01, ***p < 0.001.

Bioinformatics analysis suggested that TARS1 overexpression was associated with T stage (T1 < T2), ER(Estrogen receptor) status (positive < negative), PR (Progesterone receptors) status (positive < negative), HER-2 (Human epidermal growth factor receptor 2) status (positive > negative) in breast cancer patients, PAM50 (LumA < LumB, HER-2, Basal) and Histological type (infiltrating ductal carcinoma > infiltrating lobular carcinoma), but not N stage, M stage and Pathological stage was not relevant (**Figure 3**, **Supplementary Table 1**). Breast cancer specimens collected in our center were processed and statistically analyzed, and typical IHC images are shown in **Figure 4**. TARS1 overexpression in breast cancer was demonstrated at the mRNA and protein levels in fresh frozen tissue and paraffin-embedded tissue, respectively (**Figure 5**). <The relationship between TARS overexpression and T stage, ER status, PR status, PAM50 and Pathological stage was consistent with the raw signal results, while the relationship with N stage (N0, N2) and HER-2 status was different.

### Correlation and enrichment analyses

**Figure 6:**
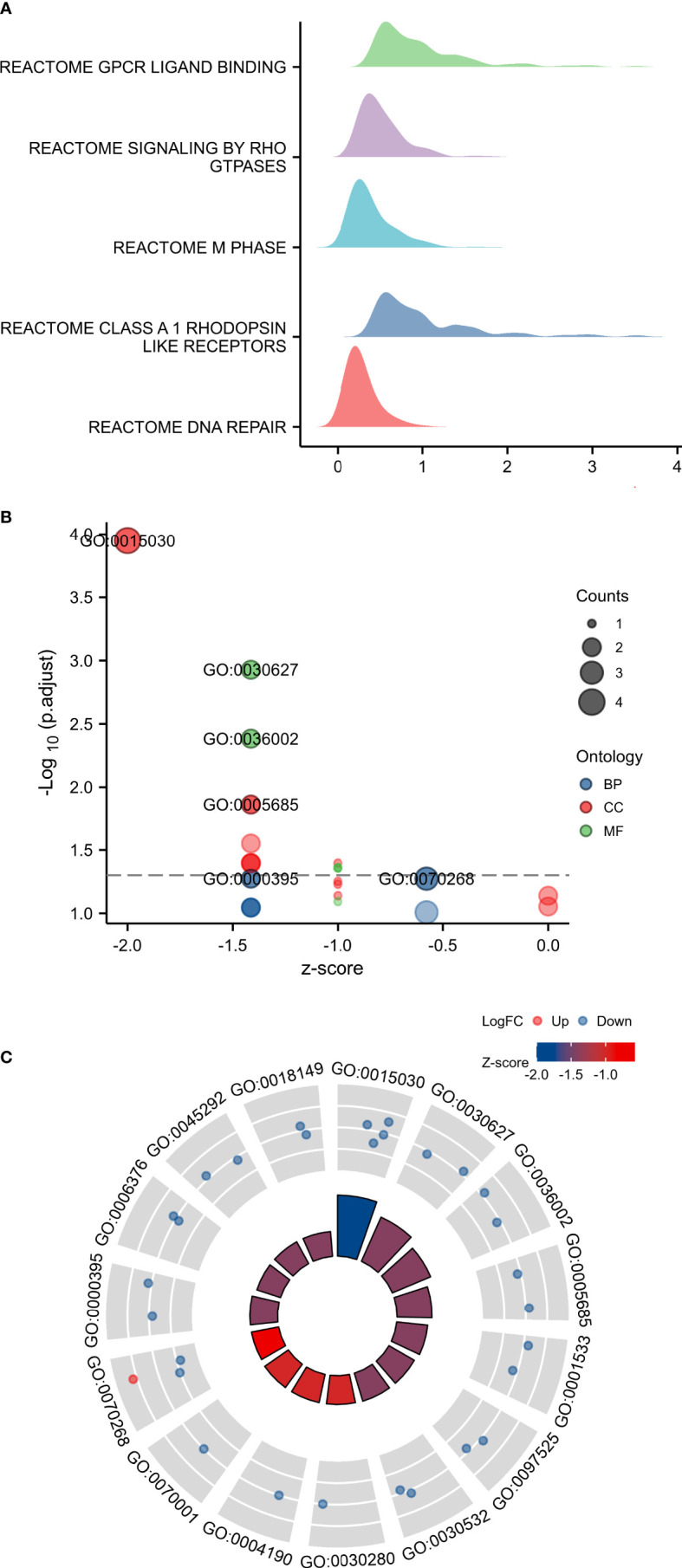
GSEA and GO/KEGG enrichment analysis of TARS1. **(A)** GSEA analysis of TARS1 **(B, C)** GO/KEGG enrichment of TARS1.

GSEA analysis of TARS1 includes GPCR ligand binding, signaling by RHO GTPases, M phase, Class A1 rhodopsin-like receptors and DNA repair (**Figure 6A**). In **Figures 6B, C**, the GO/KEGG enrichment study is displayed.

### TARS1 expression and immune cell infiltration

**Figure 7:**
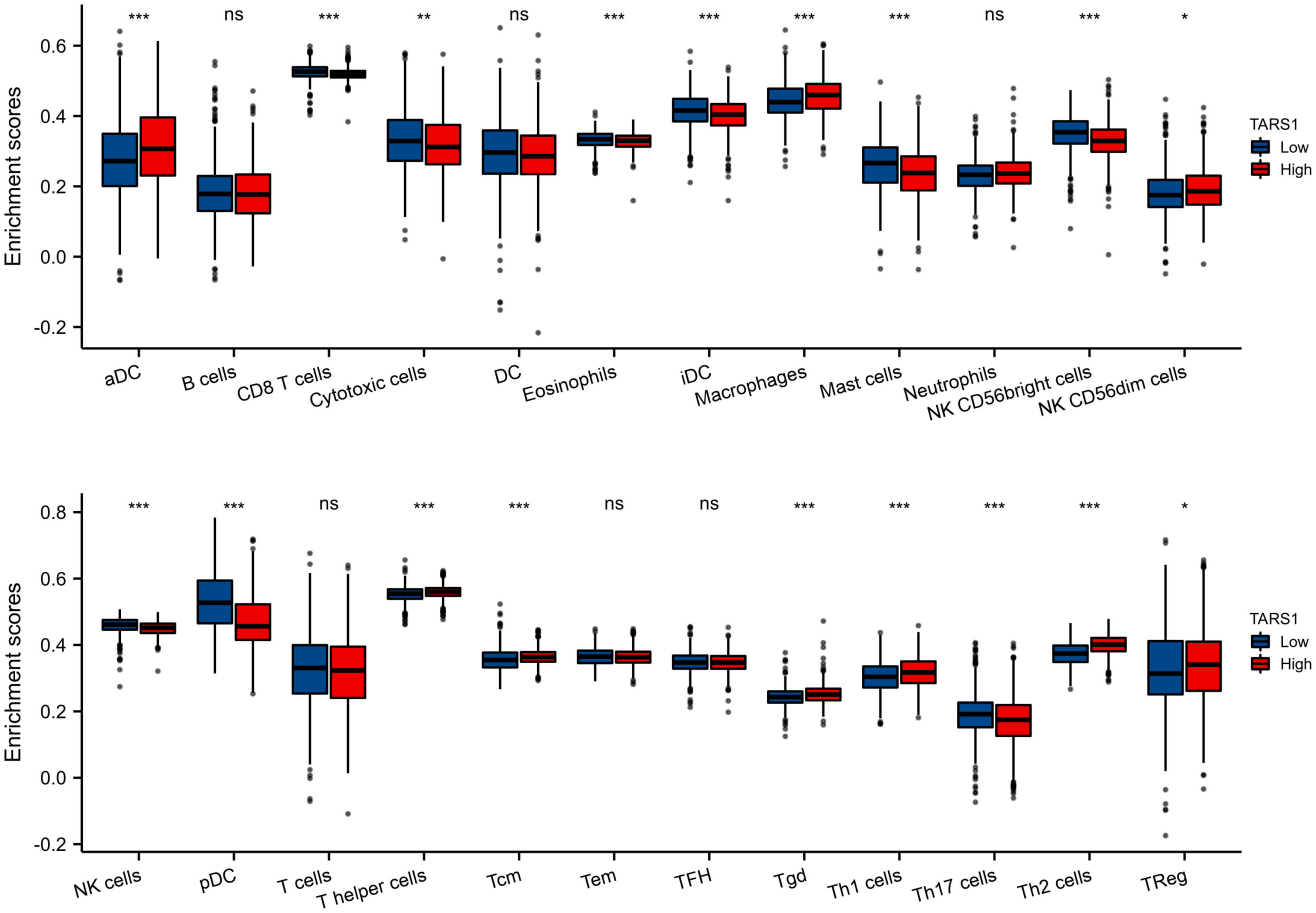
The expression level various immune cell infiltration in High and low TARS1 breast cancer. *p < 0.05, **p < 0.01, ***p < 0.001. ns, not significant.

**Figure 8:**
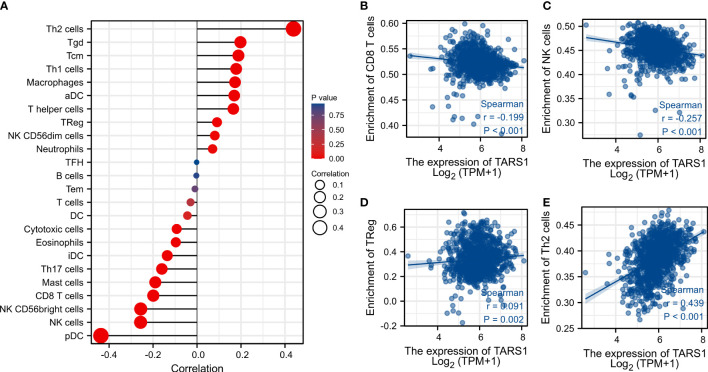
Associated between TARS1 with immune cell infiltration. **(A)** Correlation between the expression level of TARS1 and various immune cell infiltration. **(B)** Correlation between TARS1 expression and CD8+T cells. **(C)** Correlation between TARS1 expression and NK cells. **(D)** Correlation between TARS1 expression and Treg cells. **(E)** correlation between TARS1 expression and Th2 cells.

**Figure 9:**
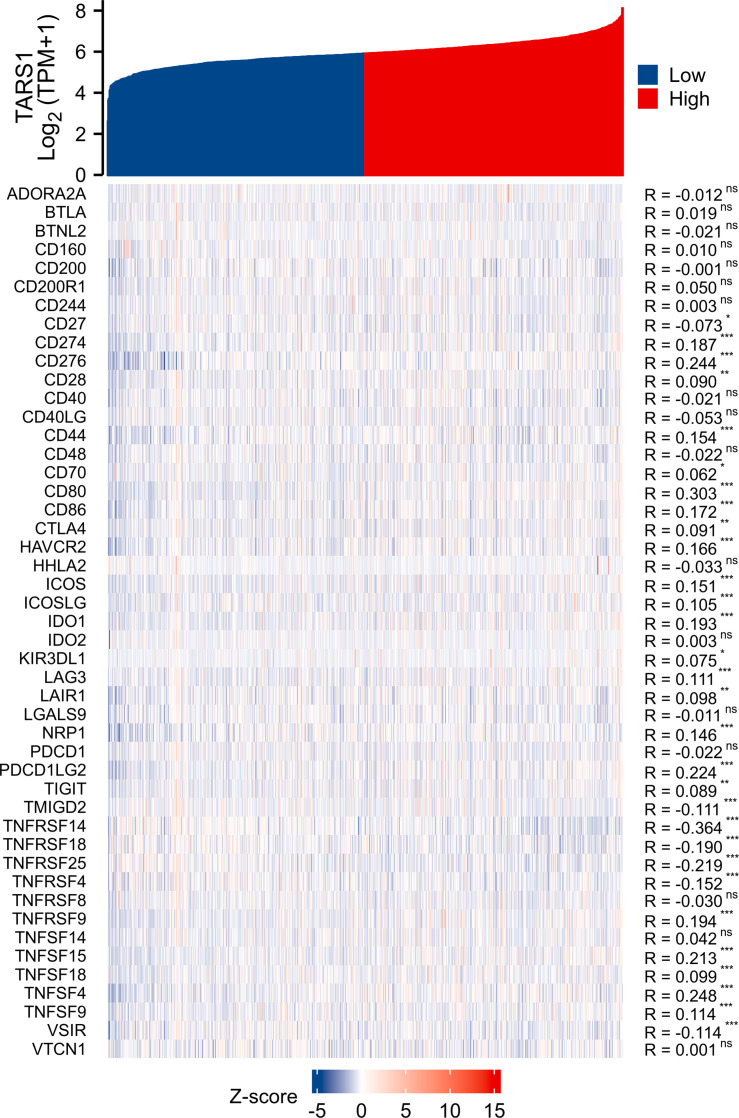
Co-expression of TARS1 and immune checkpoint related genes. *p < 0.05, **p < 0.01, ***p < 0.001. ns, not significant.

**Figure 10:**
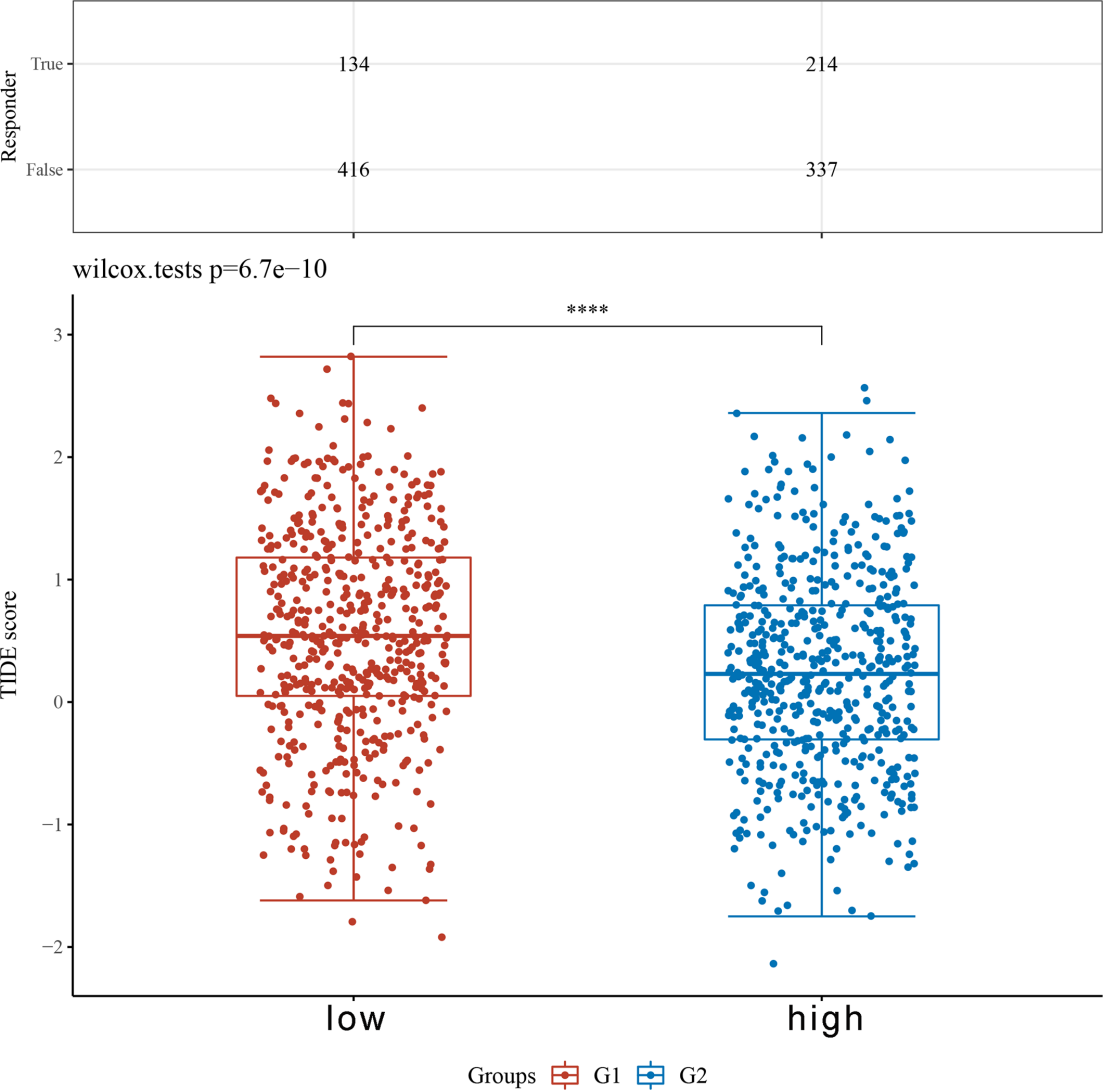
TIDE based on the expression level of TARS1. ****p < 0.0001.

Based on the median expression of TARS1, breast cancers from TCGA-BRCA Database (removed duplicate samples and those lacking clinical information) were divided into low and high expression groups and immune cell infiltration was separately analyzed. **Figure 7** shows the comparison of 24 immune cell pairs. A significant number of anti-tumor cells, such as CD8+ T cells and NK cells, were found in the TARS1 high expression group, as opposed to pro-tumor cells, such as Tregs and Th2 cells (**Figure 8**). The RNA-sequencing expression (level 3) profiles and corresponding clinical information for breast cancer (BC) were obtained from the TCGA dataset. In order to evaluate the credibility of immune score assessment, the immuneeconv R software package was utilized. This package incorporates six contemporary algorithms, namely TIMER, xCell, MCP-counter, CIBERSORT, EPIC, and quanTIseq, all of which have been benchmarked and possess distinctive strengths. In an investigation of the coexpression of TARS1 with 47 immune checkpoint-related genes, 30 were found to coexpress with TARS1. The 47 immune checkpoint-related genes frequently identified in prior studies were chosen. (**Figure 9**). The TIDE algorithm (29) additionally revealed that breast cancers with higher TARS1 expression responded better to immunotherapy with checkpoint inhibitors. (**Figure 10**).

**Figure 11:**
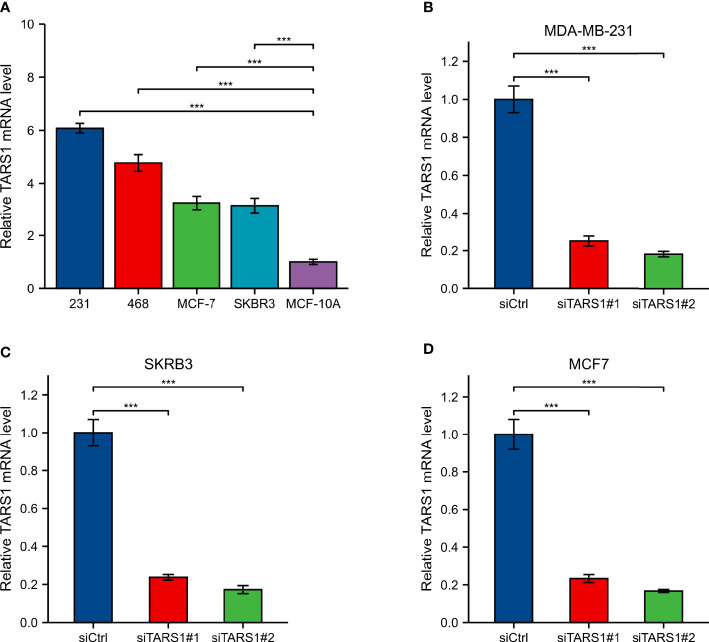
Expression and knockdown of TARS1 in various cell lines **(A)** TARS1 expression in MDA-MB-231, MDA-MB-468, MCF7, SKBRE3, and MCF10A cell lines. **(B-D)** TARS1 knockdown efficiency of two siRNA in MDA-MB-231, SKBR3, MCF7 cell lines. ***p < 0.001.

**Figure 12:**
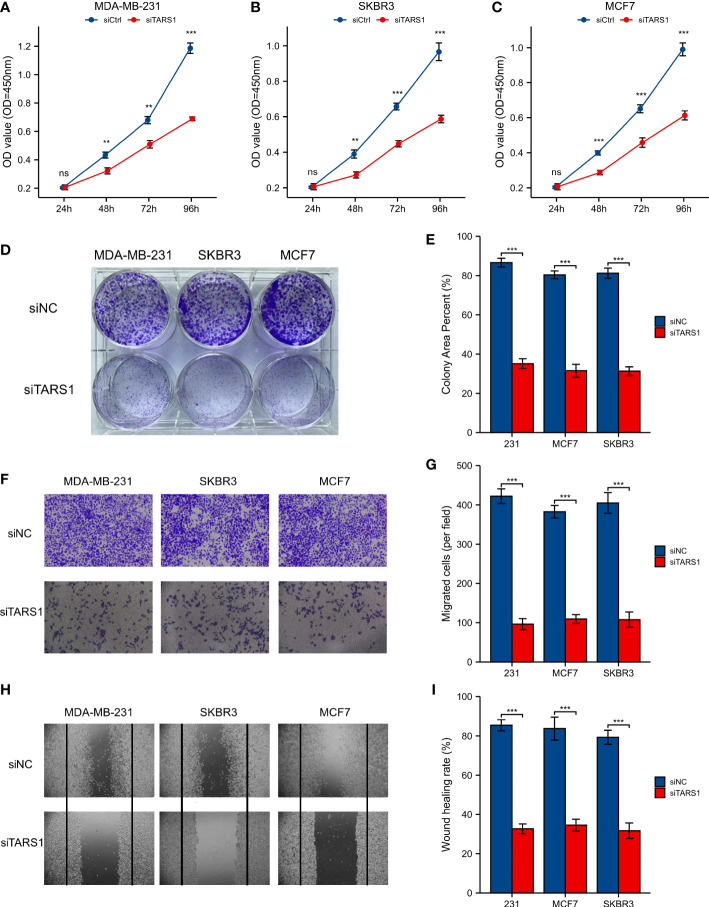
CCK8 experiment, Clone formation experiment, Transwell experiment and scratch experiment. **(A-C)** CCK-8 experiment in MDA-MB-231, SKBR3, MCF7 cell lines. **(D, E)** Clone formation of control group and two siRNA knockout groups in MDA-MB-231, SKBR3, MCF7 cell lines and quantitative analysis **(F, G)** Transwell images of control group and two siRNA knockout groups in MDA-MB-231, SKBR3, MCF7 cell lines and quantitative analysis. **(H, I)** Scratch test images of control group and two siRNA knockout groups in MDA-MB-231, SKBR3, MCF7 cell lines and quantitative analysis. All assays were independently repeated at least three times. Data are presented as the mean ± SD. **p < 0.01, ***p < 0.001, **** p < 0.0001. ns, not significant.

Breast cancer cells with TARS1 knockdown displayed reduced malignant behavior:

A significant increase in TARS1 expression was observed in MDA-MB-231, MDA-MB-468, SKBR3 and MCF-7 cells in comparison with normal mammary cells, MCF-10A (**Figure 11A**). The initial four cell lines represent three types of breast cancer: triple negative, HER-2 positive, and hormone receptor positive, while the MCF-10A cell line represents normal breast cells. TARS1 was successfully knocked down in MDA-MB-231, SKBR3 and MCF-7 cells using siRNA (**Figures 11B–D**), and CCK8 (**Figures 12A–C**) and clone formation (**Figures 12D, E**) assays revealed that breast cancer cell proliferation was significantly reduced. The Transwell assay (**Figures 12F, G**) and the scratch assay (**Figures 12H, I**), in contrast, demonstrated that the ability of breast cancer cells to invade was greatly diminished. Using ImageJ and AI software, statistically significant results were obtained from the clone formation assay, Transwell assay, and scratch assay. (**Figures 12D–F**).

## Discussion

Throughout the past four decades, breast cancer incidence has been increasing. From 2010 to 2019, the incidence of breast cancer increased on average by 0.5% each year (30). Biomarkers like ER, PR, and HER-2 play a crucial role in the diagnosis and management of breast cancer (31). However, there is still no effective treatment for triple-negative breast cancer, and hormone receptor-positive and HER-2-positive cancers also experience drug resistance (32). It is vital to discover new causative genes or therapeutic targets in order to treat breast cancer.

TARS1 is significantly expressed in a number of malignancies, including breast cancer, and is associated with poor prognoses TARS1 overexpression was linked to bigger primary tumor size, hormone receptor negativity, and HER-2 receptor positivity, according to further study of the clinicopathological characteristics of BC patients. TARS1 is overexpressed in breast cancer at both the transcriptional and translational levels, according to quantitative analysis of breast cancer specimens obtained at our center. TARS1 is a crucial constituent of mRNA translation in vertebrates and serves a significant function in protein synthesis. Previous research has shown that TARS1 is secreted in inflammatory states and stimulates endothelial cell migration and angiogenesis, and given the hypermetabolic state of tumors and their reliance on neovascularisation, researchers believe this is one of the reasons why it promotes the development of breast cancer (12).Breast cancer cells exhibit a swift metabolism and abbreviated proliferation cycle in contrast to normal breast cells, resulting in more robust protein synthesis, particularly during cellular metamorphosis and migration. Consequently, TARS1 overexpression aligns with the heightened metabolic state of cancer cells, potentially contributing to the promotion of breast cancer proliferation and migration. Breast cancer patient clinicopathological characteristics were quantified using immunohistochemistry, and it was discovered that overexpression of TARS1 was linked to higher initial tumor sizes and hormone receptor negativity. According to bioinformatic study, this is accurate. However, our results suggest that TARS1 overexpression is more pronounced in patients with lymph node metastases, independent of HER-2 expression, which is different from the bioinformatic results and requires further validation with larger volume samples. In conclusion, the association of TARS1 overexpression and clinicopathological features of breast cancer targets a possible beneficiary population for its clinical translation.

Immunotherapy brings a new light to cancer patients. It is important for stromal cells in the tumor microenvironment, particularly immune cells, to regulate tumor cell malignancy (33). They are clinically important for assessing cancer patients’ prognosis and treatment outcomes, according to a growing body of research (34, 35). In our study, we discovered that TARS1 overexpression inhibited the infiltration of anti-cancer immune cells like CD8+ T cells and NK cells while promoting the infiltration of oncogenic immune cells like Treg and Th2 in breast cancer. A drop in anti-tumor cells promotes breast cancer cell proliferation, but an increase in pro-tumor cell infiltration generates an immunological milieu more suitable for breast cancer cell migration. This could be one of the reasons why TARS1 overexpression enhances breast cancer cell proliferation and migration, ultimately leading to a poor prognosis for patients with breast cancer. KEYNOTE-086 study (36) demonstrates the safety and antitumor activity of pablizumab monotherapy in metastatic TNBC, suggesting its use as first-line treatment for mTNBC. On the basis of this, KEYNOTE-355 study (37) further demonstrate that for metastatic TNBC with CPS ≥10, pablizumab in combination with chemotherapy improved progression-free survival significantly more than placebo in combination with chemotherapy suggests that adding pablizumab to standard chemotherapy in the first-line treatment of metastatic triple-negative breast cancer is important. As the indications for immune checkpoint inhibitors expand, the question of how to find the patients most likely to benefit and accurately predict efficacy has become a concern. A majority of immune checkpoint-associated genes are co-expressed with TARS1, implying that it may be inter-regulated with multiple targets in the immune checkpoint-associated pathway and could be a predictive biomarker of efficacy or a novel therapeutic target for ICI in the treatment of BC. In the TIDE algorithm, which assesses tumor immune escape by using several gene expression markers, two mechanisms are assessed, A number of immunosuppressive factors, as well as dysfunction of tumor-infiltrating cytotoxic T lymphocytes (CTL), were used as a means of testing our suspicions (38). It has been shown that the higher the TIDE score, the less effective immune checkpoint blockade therapy (ICB) is and the shorter the survival after ICB. TARS1 high expression groups were found to be more responsive to ICB.

Overall, our study found that TARS1 may be a causative gene in breast cancer and is more significantly overexpressed in breast cancers with specific clinicopathological features. TARS1 overexpression leads to a suppressed state of immune infiltration within breast cancer and may be a predictive biomarker for the efficacy of immunotherapy in breast cancer.

## Data availability statement

The original contributions presented in the study are included in the article/**Supplementary Material**. Further inquiries can be directed to the corresponding author.

## Ethics statement

A protocol for this study has been approved by the Ethics Committee of Tongji Hospital in accordance with the Helsinki Declaration (approval number TJIRB20221218).

## Author contributions

ZG and PL obtained clinical specimens; PL performed the data analysis; DZ performed the formal analysis; ZG and DZ performed immunohistochemical analysis; WW and PL wrote the manuscript. All authors contributed to the article and approved the submitted version.
