# Supplementary material for: Clinical implications and immune implications features of TARS1 in breast cancer
Source: Front Oncol. 2023 Aug 11;13:1207867. doi: 10.3389/fonc.2023.1207867 (PMC10455957; doi:10.3389/fonc.2023.1207867)
Supplement: Supplementary file 1 [file Table_1.docx]

Clinicopathologic variables associated with TARS1 expression

| Characteristic | Low expression of TARS1 | High expression of TARS1 | p |
| --- | --- | --- | --- |
| n | 541 | 542 |  |
| T stage, n (%) |  |  | 0.026 |
| T1 | 151 (14%) | 126 (11.7%) |  |
| T2 | 302 (28%) | 327 (30.3%) |  |
| T3 | 76 (7%) | 63 (5.8%) |  |
| T4 | 11 (1%) | 24 (2.2%) |  |
| N stage, n (%) |  |  | 0.049 |
| N0 | 256 (24.1%) | 258 (24.2%) |  |
| N1 | 187 (17.6%) | 171 (16.1%) |  |
| N2 | 45 (4.2%) | 71 (6.7%) |  |
| N3 | 43 (4%) | 33 (3.1%) |  |
| M stage, n (%) |  |  | 0.360 |
| M0 | 432 (46.9%) | 470 (51%) |  |
| M1 | 7 (0.8%) | 13 (1.4%) |  |
| Pathologic stage, n (%) |  |  | 0.498 |
| Stage I | 98 (9.2%) | 83 (7.8%) |  |
| Stage II | 312 (29.4%) | 307 (29%) |  |
| Stage III | 117 (11%) | 125 (11.8%) |  |
| Stage IV | 7 (0.7%) | 11 (1%) |  |
| Race, n (%) |  |  | 0.217 |
| Asian | 25 (2.5%) | 35 (3.5%) |  |
| Black or African American | 93 (9.4%) | 88 (8.9%) |  |
| White | 401 (40.3%) | 352 (35.4%) |  |
| Age, n (%) |  |  | 0.345 |
| <=60 | 292 (27%) | 309 (28.5%) |  |
| >60 | 249 (23%) | 233 (21.5%) |  |
| Histological type, n (%) |  |  | < 0.001 |
| Infiltrating Ductal Carcinoma | 333 (34.1%) | 439 (44.9%) |  |
| Infiltrating Lobular Carcinoma | 155 (15.9%) | 50 (5.1%) |  |
| ER status, n (%) |  |  | < 0.001 |
| Negative | 83 (8%) | 157 (15.2%) |  |
| Indeterminate | 1 (0.1%) | 1 (0.1%) |  |
| Positive | 434 (41.9%) | 359 (34.7%) |  |
| PR status, n (%) |  |  | < 0.001 |
| Negative | 135 (13.1%) | 207 (20%) |  |
| Indeterminate | 1 (0.1%) | 3 (0.3%) |  |
| Positive | 382 (36.9%) | 306 (29.6%) |  |
| HER2 status, n (%) |  |  | 0.001 |
| Negative | 286 (39.3%) | 272 (37.4%) |  |
| Indeterminate | 5 (0.7%) | 7 (1%) |  |
| Positive | 55 (7.6%) | 102 (14%) |  |
| PAM50, n (%) |  |  | < 0.001 |
| Normal | 30 (2.8%) | 10 (0.9%) |  |
| LumA | 357 (33%) | 205 (18.9%) |  |
| LumB | 72 (6.6%) | 132 (12.2%) |  |
| Her2 | 19 (1.8%) | 63 (5.8%) |  |
| Basal | 63 (5.8%) | 132 (12.2%) |  |
| Menopause status, n (%) |  |  | 0.454 |
| Pre | 106 (10.9%) | 123 (12.7%) |  |
| Peri | 20 (2.1%) | 20 (2.1%) |  |
| Post | 359 (36.9%) | 344 (35.4%) |  |
| Anatomic neoplasm subdivisions, n (%) |  |  | 0.121 |
| Left | 268 (24.7%) | 295 (27.2%) |  |
| Right | 273 (25.2%) | 247 (22.8%) |  |
| radiation_therapy, n (%) |  |  | 0.829 |
| No | 226 (22.9%) | 208 (21.1%) |  |
| Yes | 283 (28.7%) | 270 (27.4%) |  |
| Age, median (IQR) | 59 (49, 68) | 57 (48, 66) | 0.098 |
